# Early surgery? In-house mortality after proximal femoral fractures does not increase for surgery up to 48 h after admission

**DOI:** 10.1007/s40520-023-02406-x

**Published:** 2023-05-04

**Authors:** Annabel Fenwick, Michael Pfann, Iana Antonovska, Jakob Mayr, Leonhard Lisitano, Stefan Nuber, Stefan Förch, Edgar Mayr

**Affiliations:** 1 Department of Trauma, Orthopedic, Plastic and Hand Surgery, University Hospital of Augsburg, Stenglinstrasse 2, 86156 Augsburg, Germany; 2 Zentrum für Unfallchirurgie und Orthopädie, Klinikum Ingolstadt GmbH, Krumenauerstraße 25, 85049 Ingolstadt, Germany

**Keywords:** Complications, Early surgery, Proximal femur fracture, Mortality rate

## Abstract

**Purpose:**

The economic cost linked to the increasing number of proximal femur fracture and their postoperative care is immense. Mortality rates are high. As early surgery is propagated to lower mortality and reduce complication rates, a 24-h target for surgery is requested. It was our aim to determine the cut-off for the time to surgery from admission and therefore establish a threshold at which the in-house mortality rate changes.

**Methods:**

A retrospective single-center cohort study was conducted including 1796 patients with an average age of 82.03 years treated operatively for a proximal femoral fracture between January 2016 and June 2020. A single treatment protocol was performed based on the type of anticoagulant, surgery, and renal function. Patient data, surgical procedure, time to surgery, complications, and mortality were assessed.

**Results:**

In-house mortality rate was 3.95%, and the overall complication rate was 22.7%. A prolonged length of hospital stay was linked to patient age and occurrence of complications. Mortality is influenced by age, number of comorbidities BMI, and postoperative complications of which the most relevant is pneumonia. The mean time to surgery for the entire cohort was 26.4 h. The investigation showed no significant difference in mortality rate among the two groups treated within 24 h and 24 to 48 h while comparing all patients treated within 48 h and after 48 h revealed a significant difference in mortality.

**Conclusions:**

Age and number of comorbidities significantly influence mortality rates. Time to surgery is not the main factor influencing outcome after proximal femur fractures, and mortality rates do not differ for surgery up to 48 h after admission. Our data suggest that a 24-h target is not necessary, and the first 48 h may be used for optimizing preoperative patient status if necessary.

## Introduction/Background

The incidence of proximal femoral fractures is rising with an increasing age of population [1, 2]. Patients often lose their pre-existing mobility and due to the subsequent impairments cannot return to daily life activities [3]. One-year postoperative mortality is up to 30% [4].

The economic costs linked to the fracture and postoperative care are immense and starting to exceed resources. The associated economic burden on the health system has led to an increased pressure on hospitals and to rising demand for early surgery for proximal femoral fractures and even same-day surgery or fast track care [5–7]. The required 24-h target can often not be met. Some countries have gone as far as to cut payments for hospitals that do not manage to meet this target [8]. But the question remains whether it is medically necessary and in the best patient’s interest to enforce surgery within 24 h even if it means operating during nighttime and/or surgeons with less experience or fatigued.

“Early surgery” has been propagated as the most influential factor determining the outcome for proximal femur fractures [9–14]. But what does “early surgery” actually mean? Literature differs from 24 h to surgery up to 4 days after admission being classified as early surgery [11, 15, 16].

It was our aim to determine the cut-off for the time to surgery from admission and therefore establish a threshold at which mortality rates change and the risk for patients rises.

## Materials and methods

### Data acquisition

For the undertaken retrospective single-center cohort study, evidence Level III, all patients treated operatively for a proximal femoral fracture (femoral neck, pertrochanteric, and sub-trochanteric fractures) at our level I trauma center during January 2016 and June 2020 were evaluated. Due to a possible bias by the COVID-19 pandemic, patients treated after June 2020 were not further included in the study. Exclusion criteria were: primary conservative treatment, greater trochanteric fractures, peri-prosthetic fractures as well as transfers for revision surgery, and poly-trauma patients.

The conducted study was approved by the Local Ethics Committee and fulfilled the standards of the declaration of Helsinki (20-2155-101).

The charts were reviewed for demographic data, such as age, gender, BMI, comorbidities including Charlson Comorbidity Index (CCI) [17], and ASA classification (American Society of Anaesthesiologists) [18], as well as fracture morphology, co-geriatric management, medication especially anticoagulants, revision surgery, and concomitant fractures. If admitted again during the above-mentioned timeframe for the contralateral side, patients were included again as a separate case. Type of surgery and especially time to surgery from admission, length of stay on intensive care unit (ICU), as well as overall length of hospital stay LHS were analyzed. Complications were recorded and divided into urinary infections, pneumonia, embolism or thrombosis, haematoma, wound infections, and mechanical complications i.e., postoperative fracture, dislocation or, cutting-out. In-house mortality and cause were evaluated.

### Therapy

The same therapy protocol existed for the total period reviewed. Aimed time to surgery was within 24 h of admission to ER for all patients without anticoagulation or only anti-platelet therapy AP including dual AP therapy. Patients with direct anticoagulants (Edoxaban, Rivaroxaban, Apixaban) were divided into two groups according to their kidney function (Gr 1: GFR > 50, Gr 2: GFR < 50). If renal clearance was good, surgery was performed within 24 h. If renal function was impaired, surgery was postponed to 24–48 h after admission to reduce risk of bleeding. For patients with Dabigatran, the time limits were prolonged to 36 and 72 h. All patients with Warfarin therapy were closely monitored and supplemented with intravenous Vit K if possible (Exceptions: Mechanical valve, thromboembolic event within last 3 months, congestive heart failure, and EF < 20%). Surgery could go ahead as the Quick value overcame 60%. No switching or bridging was done preoperatively. PPSB was not administered.

According to pre-operative mobility and comorbidities as well as fracture morphology, total or hemi-arthroplasty (cemented or uncemented, Fa. Zimmer Biomet, Indiana, US) was performed for femoral neck fractures, intramedullary nailing PFNa, Fa. Synthes, Oberdorf, Switzerland, (± cerclage) for pertrochanteric fractures and plate/screw osteosyntheses for undisplaced pertrochanteric or lateral femoral neck fractures. All sub-trochanteric fractures were addressed by open reduction, cerclage, and intramedullary nailing in side- positioning. 30 min prior to surgery, all patients received an i.v. single shot of 2 g Cefazolin.

Postoperatively, venous thromboembolism prophylaxis was given from day one with Enoxaparin 40 mg subcutaneously. Anticoagulants were substituted with Innohep according to patient weight. All patients were allowed full weight-bearing immediately after surgery and received physiotherapy from day one. Labs were taken on the first, 4th–6th day postoperatively to determine blood loss. Hemoglobin levels under 7 g/l received blood transfusions if consented. Between 7 and 8 g/l, transfusions were done depending on symptoms and cardiovascular risk factors.

### Statistical analysis

Statistical analysis was carried out with IBM SPSS Statistics (version 27; IBM Deutschland Ltd., Ehningen, Germany). Normal distribution of all data was verified. The Student’s t test, chi-square, ANOVA variance, and multivariate analysis were used to determine differences and influencing factors regarding complications and mortality. 95% confidence intervals and standard deviations were calculated. For data without normal distribution, the Wilcoxon Rank Test was used. We used the Fisher’s exact test for description of significant differences in mortality between the groups. Survival analysis was shown with Kaplan–Meier curves. The Cox regression was used to eliminate the influence of confounders for each examined variate. The significance level was set at 5% (α = 0.05).

## Results

### Demographic data

1796 patients were included and were predominantly female (distribution: female: 71.1%; male: 28.9%). The average age was 82.03 years (range: 60 – 103; SD 8.8). Patients younger than 60 years were excluded to avoid false mortality rates. The mean BMI was 24.38 kg/m^2^ (range: 11.7–66 kg/m^2^).

The cohort comprised 806 femoral neck fractures, 869 pertrochanteric fractures, and 121 sub-trochanteric fractures. In 355 cases, a total hip endo-prosthesis was implanted. 442 patients received a hemiarthroplasty. Intramedullary nailing was performed in 980 cases, 14 patients obtained dynamic hip screw (DHS) and 5 screw or plate osteosynthesis. The fracture types showed no differences for demographic data except age.

**Fig. 1:**
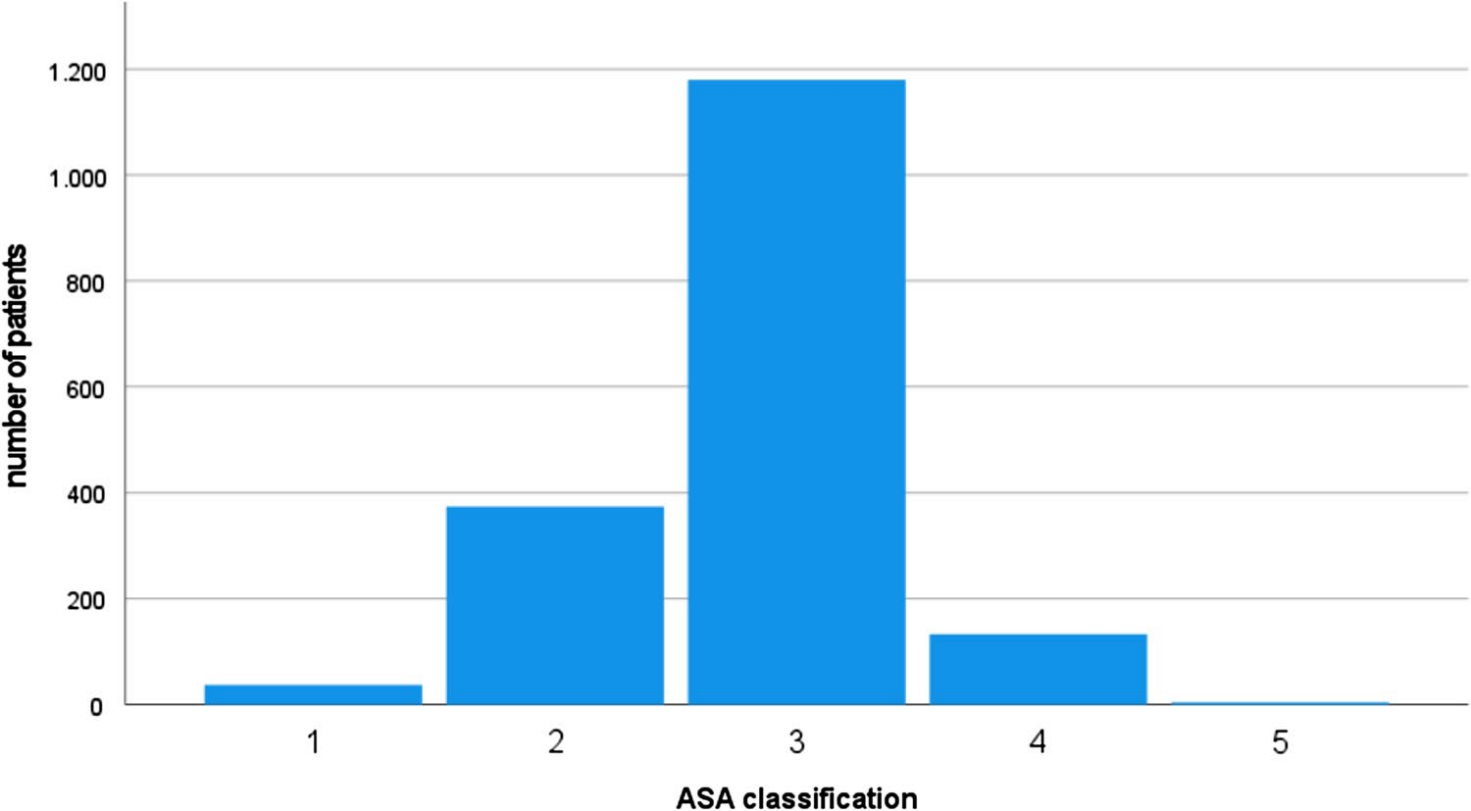
Distribution of ASA classification

Analysis of comorbidities demonstrated a mean Charlson Comorbidity Index (CCI) of 6.21 points (range: 2–15, SD: 2.2). Most patients were preoperatively classified as ASA II or III. The exact distribution is shown in Fig. 1.

Administration of rheological medication was recorded for 50.6% of the cohort at the time of admission. Of these patients, 220 patients were on DOACs (Edoxaban, Rivaroxaban, Apixaban, Dabigatran). A further 116 patients were admitted on Warfarin. Another 563 patients received anti-platelet therapy including monotherapy or dual anti-platelet inhibition, and 3 patients were on Tinzaparin.

### Length of hospital stay, LHS

The average length of hospital stay was 13.95 days (range: 1–83; SD 7.1) and the average time on the intensive care unit was 0.52 days (range: 0–23; SD 1.5). There was a positive correlation with the number of comorbidities CCI and LHS (*p* < 0.000; *R* = 0.155). Surgical procedures did not differ in LHS. But the occurrence of complications was linked to a prolonged hospitalization. Furthermore, LHS was linked to the patient age (*p* < 0.089).

### Complications and mortality

**Fig. 2:**
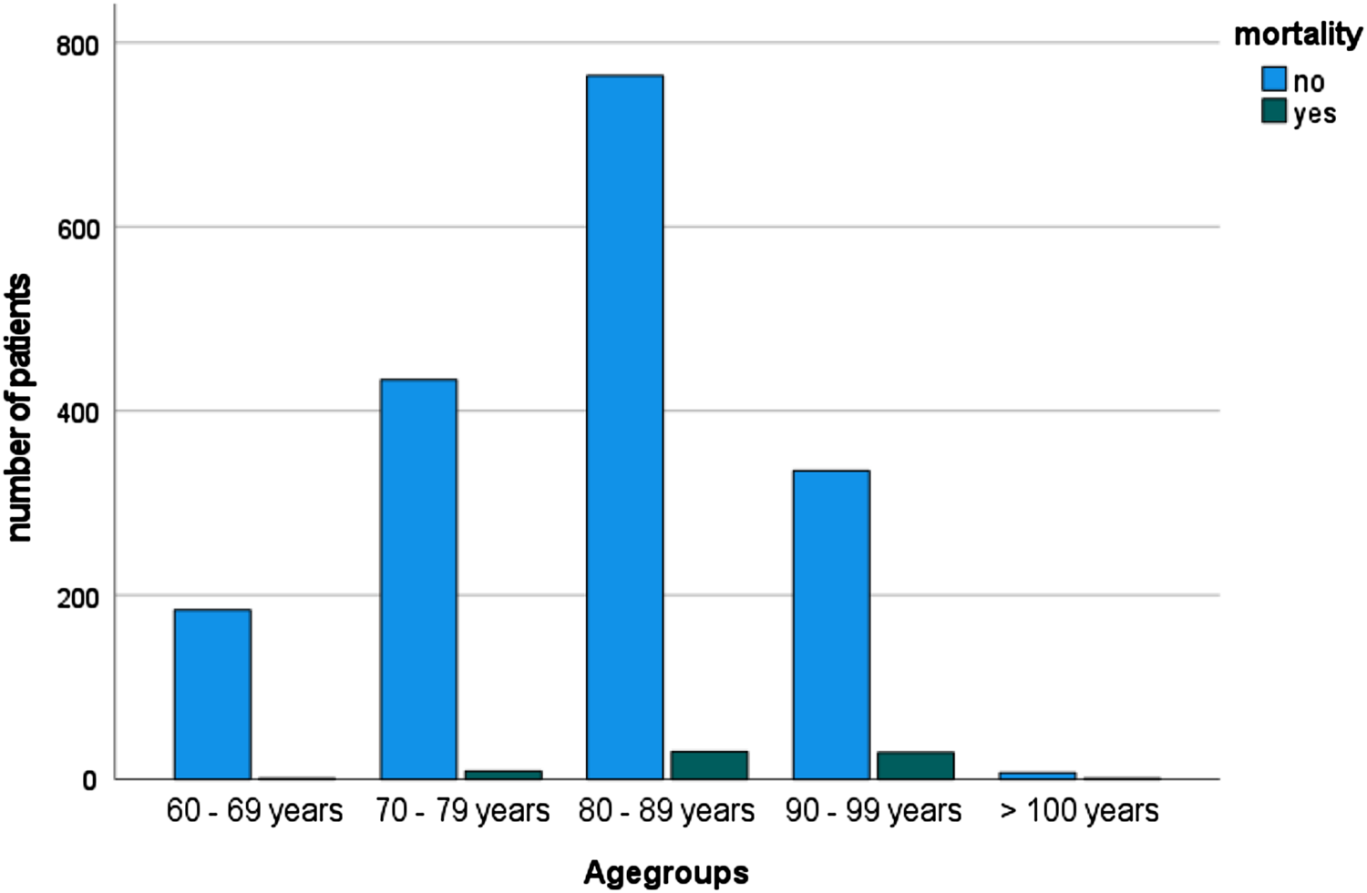
Distribution of mortality within age groups

71 of 1796 patients died during the hospital stay, which reveals a total in-house mortality rate of 3.95%. Causes for death were pulmonary embolism, cement reaction/embolism intraoperatively, cardiac arrest, and fulminant sepsis. Deceased patients had significantly more comorbidities (CCI: 6.15 vs. 7.59 points; SD2.1; *p* < 0.001) and were all grouped ASA III and IV preoperatively. Cox regression shows the influence of preoperative patient status rated by Charlson Comorbidity index (odds ratio: 1.235; confidence interval: 1.092–1.396; *p* < 0.001). Furthermore, age is a predominant factor for mortality. Patients were grouped according to their age (1: 60–69y, 2: 70–79y, 3: 80–89y, 4: 90–99y, 5: > 100y). The significance of age on mortality is demonstrated in the performed Cox regression (odds ratio: 1.644; confidence interval: 1.1173 – 2.305; *p* < 0.004) seen in Fig. 2. While sex has no effect, BMI showed an influence on mortality (odds ratio: 0.923; confidence interval: 0.867 – 0.983; *p* < 0.013).

The overall complication rate was 22.7%. Patients with complications showed a higher mortality rate in comparison to those without (8.8% vs. 2.5%; *p* < 0.000). Focusing on the main major complications, we performed a Cox regression analysis to investigate the influence on the mortality rate. Pneumonia is the most relevant complication associated with postoperative mortality in our cohort. Out of 109 patients with pneumonia, 21.2% died (odds ratio 0.238; confidence interval: 0.136–0.416; *p* < 0.000). Urinary tract infections did not have a significant influence on mortality (odds ratio 1.699; confidence interval: 0.795–3.631; *p* < 0.171). The same applies to postoperative hematoma, which did not show a difference in mortality rate (4.7% vs. 3.9%) and deep wound infection, of which there were no cases among the patients who died (*p* < 0.966).

### Time to surgery

**Fig. 3:**
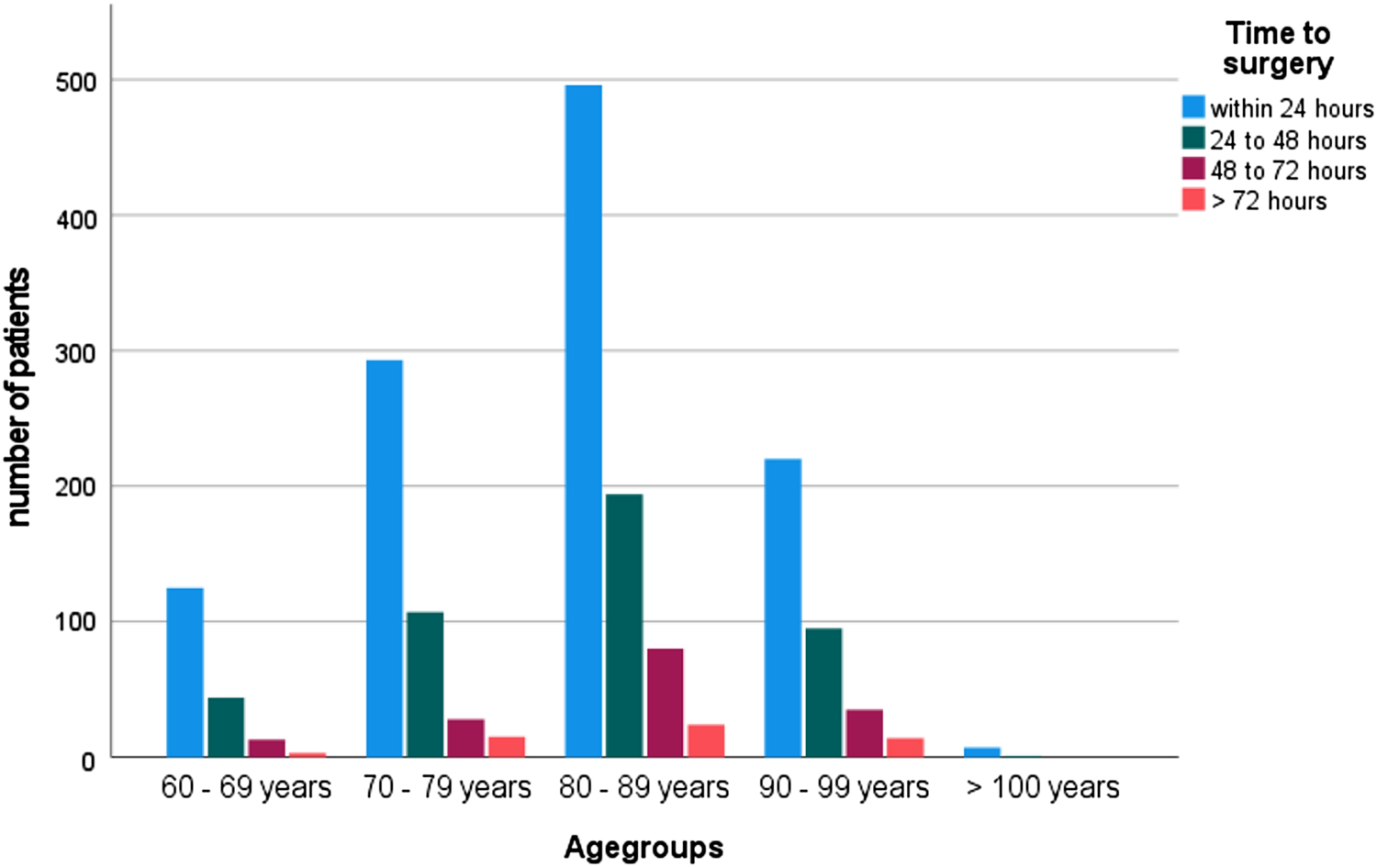
Time to surgery according to age groups

**Fig. 4:**
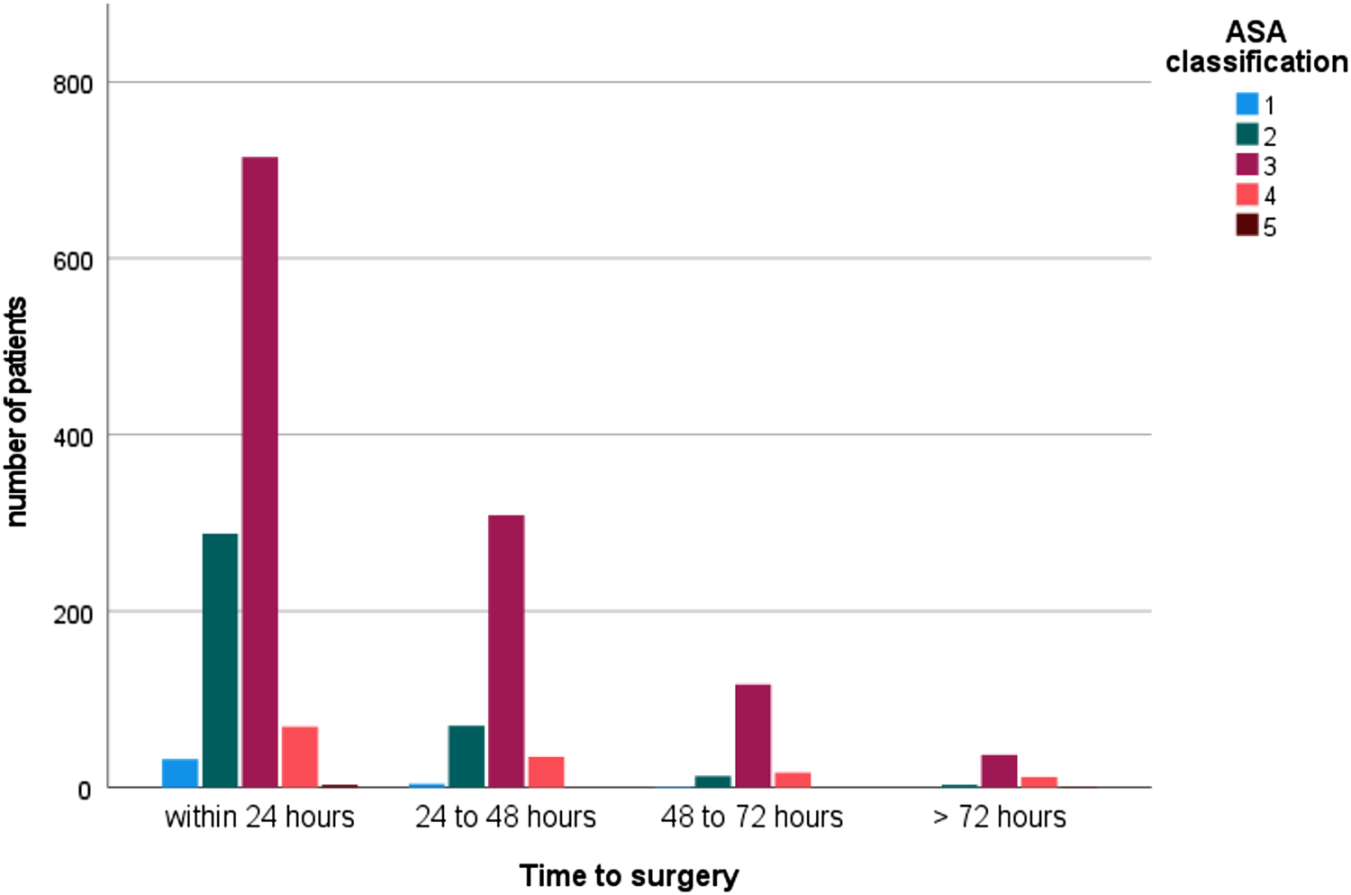
Preoperative patient status (ASA classification) grouped by time to surgery

**Fig. 5:**
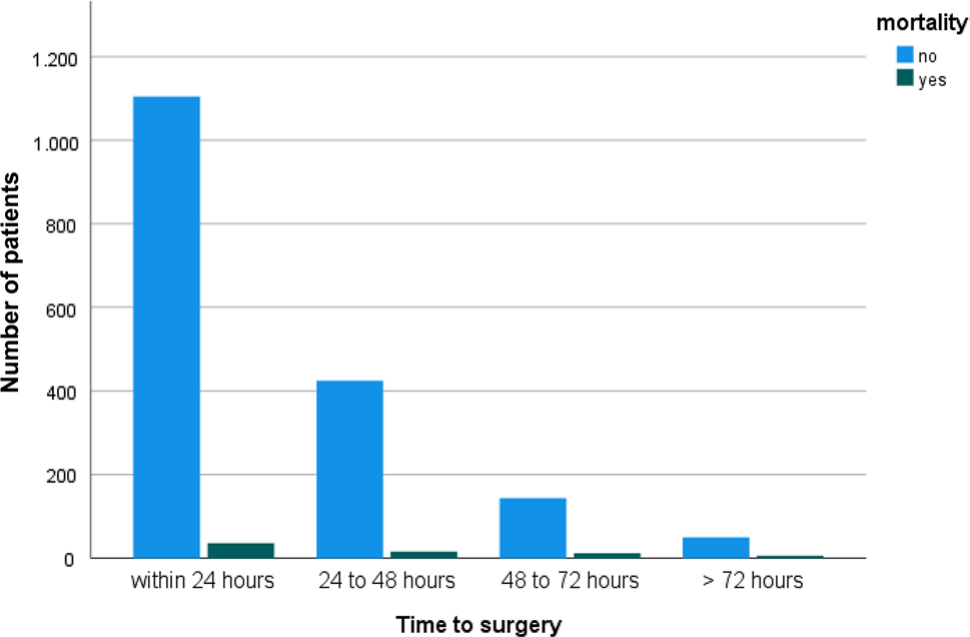
Mortality depending on time to surgery from admission

**Fig. 6:**
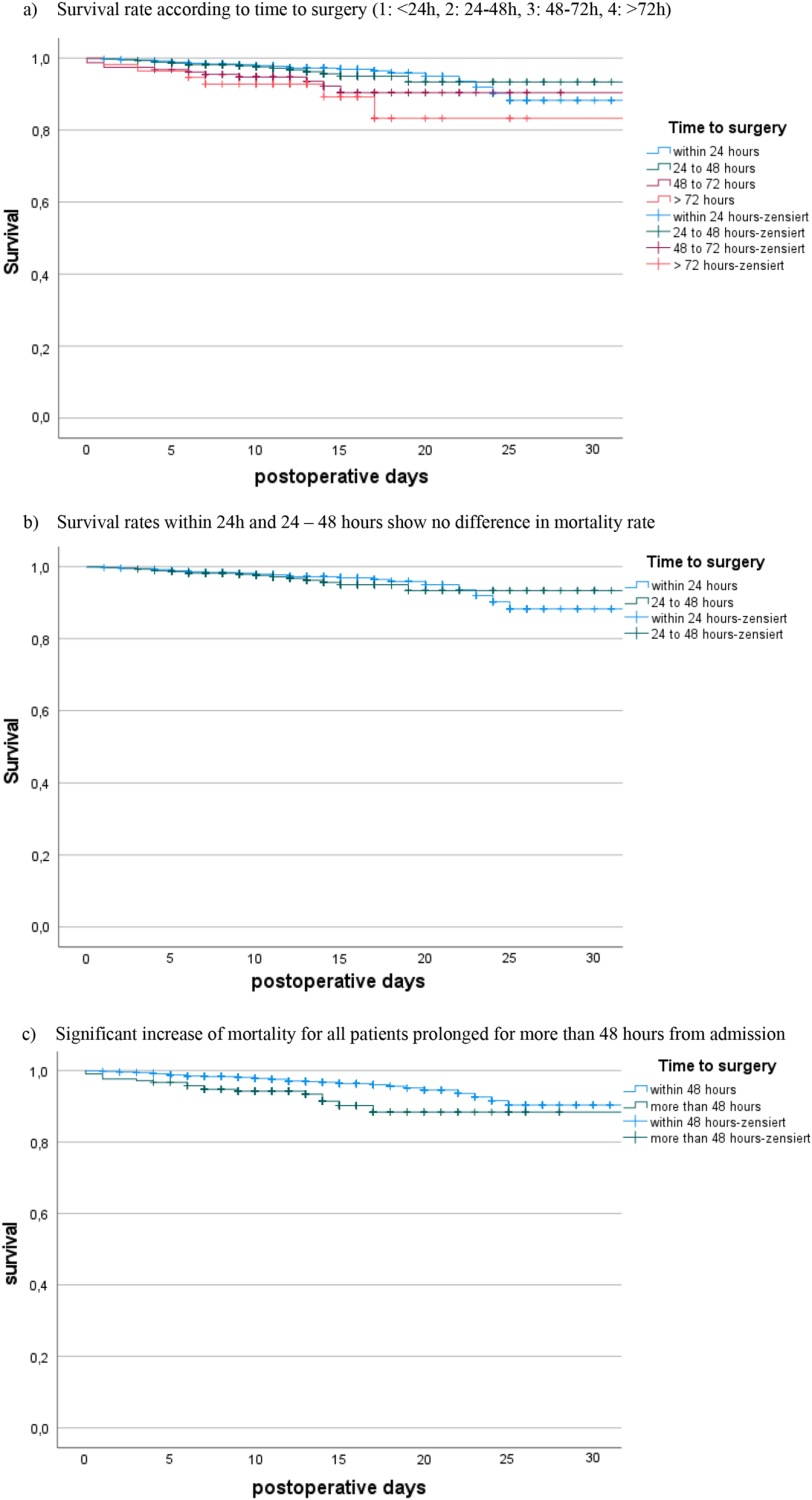
Survival rates (Kaplan–Meier)

The mean time to surgery for the entire cohort was 26.4 h (range: 0.95–256.8; SD: 20.2) from admission. We initially divided the patients into subgroups depending on the time to surgery (1: within 24 h, 2: 24–48 h, 3: 48–72 h and 4: more than 72 h). The mentioned subgroups demonstrated a difference in age and number of comorbidities as seen in Table 1 and Fig. 3. All patients treated within 48 h had less comorbidities than patients with surgery prolonged for more than 48 h, Fig. 4. 63.53% of the cohort had surgery within 24 h of admission and 88.08% could be treated within 48 h. Main reasons for delay were infection at time of admission in need of i.v. antibiotics and limited operating room (OR) or ICU capacity. On average, patients who died showed a longer wait for surgery (36.22 h; SD 35.89 h vs. 25.99 h; SD 19.22 h). Figure 5 shows the exact distribution among the established groups. The subgroup operated on within 24 h had the lowest mortality rate of all the groups (p < 0.014); thus, more detailed investigation showed no significant difference of mortality rate among the two groups treated within 24 h and 24 to 48 h (*p* < 0.906). The comparison between all patients treated within 48 h with all patients with surgery prolonged for more than 48 h reveals a significant difference in mortality rate (p.002), Fig. 6.

**Table 1:** Demographic data for groups grouped by time to surgery from admission

	Within 24 h	24 to 48 h	48 to 72 h	More than 72 h
Number of patients	1141	441	156	56
Age	81.73 (SD 8.9)	82.37 (8.7)	83.56 (SD 7.8)	82.93 (SD 9.03)
Gender (female)	829	313	107	35
BMI in kg/m^2^	24.17 (SD 4.39)	25.02 (SD 4.95)	24.73 (SD 4.82)	24.35 (SD 4.48)
CCI in points	6.06 (SD 2.19)	6.32 (SD 2.16)	6.79 (SD 2.06)	6.67 (SD 2.00)
Mortality rate in %	3.15 (*N* = 36)	3.62 (*N* = 16)	7.69 (*N* = 12)	10.7 (*N* = 6)

## Discussion

It is a given fact that patients suffering from a hip fracture have an increased mortality risk during the following year [3]. Various factors have been determined. Thus, the question remains how influential time to surgery is and where the threshold should be drawn. Very few prospective studies, but mostly retrospective studies have been conducted often only involving a small number of patients while larger studies are often meta-analysis including multiple centers with inhomogeneous groups and different approaches.

Welford [19] analyzed 46 studies from January 1991 to June 2020 comprising 521,857 hip fractures with 64,047 postoperative deaths. His analysis concluded that surgery within 24 h led to lower mortality than surgery after 24 h after admission.

In contrast to those findings, another large systematic review of 52 published studies involving 291 143 patients which was recently performed by Khan et al. observed that when studies adjusted for confounding factors, they were less likely to report improved survival outcomes by early surgery [13].

“Early surgery” remains an inconsistent term. Moran et al. [16] defined “early surgery” as being surgery on the day of admission and the day after. When he compared the mortality rates, he did not find increased mortality up to four days after admission. His long-term data identified pneumonia as a risk factor linked to a higher mortality rate 90 days and one year post operatively.

Various studies have compared different waiting times to surgery. Uzoigwe et al. [11] reported their results of 2056 patients operated within 12, 24, and after 36 h. The authors found increased mortality after 36 h and progressively decreased mortality in patients operated within 24 and 12 h while Al-Ani et al. [10] reported no significant difference in mortality rates at either 24, 36, or 48 h. Grimes et al. showed no increase in long-term mortality rates even for delays for more than 96 h [20].

Maheshwari et al. investigated 720 patients with hip fractures und concluded that each hour of delay from admission to surgery was associated with a 5% higher risk of one-year postoperative mortality [21].

Mortality is certainly influenced by time to surgery [22]. But is timing the main factor influencing mortality? Copious studies have focused on just this, on the other hand, investigations have tried to define other important factors. Fröhlich et al. [23] claim that comorbidities and age over 75 years are associated with a higher mortality one year postoperatively. Another study also demonstrated preexistent comorbidities evaluated by CCI to be the determining factor for short- and long-term mortalities after hip fractures [24]. In agreement, Schwitzer et al. [25] concluded that time to surgery from admission did not affect 30-day mortality or the total number of perioperative complications while age, ASA score, and CCI were associated with adverse outcomes.

Simunovic et al. [15] performed a further meta-analysis and concluded that early surgery was beneficial as associated with lower mortality rates and reduced the risk of pneumonia and pressure sores. But in this study, early surgery was defined as within 72 h. The authors further adjusted for confounding preoperative factors such as underlying illnesses, which reduced the effect but still showed a trend toward reduced mortality.

A rare prospective design study was carried out by Smektala et al. including 246 acute care hospitals in Germany investigating one-year mortality for proximal femur fractures. The results showed no effect of time to surgery on the mortality rate. The early group had a reduction of postoperative complications, such as pneumonia und urinary infections, but a higher risk of bleeding- or implant-associated complications [26]. This is supported by an autopsy study showing surgery within 24 h significantly reduced death by pneumonia and pulmonary embolism [27].

Our results suggest that surgery within 48 h is safe and does not increase mortality rates. This is supported by a study by Leer–Salvesen reporting about the Norwegian Hip Fracture Register [28]. The cohort comprises 83,737 hip fractures. The authors agree with our findings that mortality rises after a total delay including the time to admission (mean of six hours) of more than 48 h. Similar results are shown by Bennett et al. [29] concluding that delaying surgery for more than 48 h, especially for multi-morbid patients, is associated with not only a rise of mortality rate but also leads to a prolonged hospital stay.

A different approach is propagated by Aprato. As early surgery for proximal femoral fractures became a priority in health systems, they emphasized early postoperative physiotherapy to play an important role in reducing mortality. Independent of time for surgery, they were able to prove that patients who did not walk again within 10 days after surgery had a higher mortality [30].

Statistical analysis of mortality after proximal femur fractures comparing early surgery to late surgery will always show the advantage of earlier surgery, but the importance is to establish the threshold from which complications and especially mortality significantly increases.

The limitations of this study are the retrospective design and the lack of long-term data, such as a one-year mortality rate. We are lacking randomized control studies which would offer more definitive evidence on the influence of surgical timing, but ethical considerations in designing such trials prevent their use. Furthermore, our anticoagulant therapy protocol for surgery leads to a certain bias by postponing surgery for more than 48 h. But still the conclusion for safe surgery up to 48 h can be clearly drawn from our data.

### Conclusion

Time to surgery as the most predominant factor influencing postoperative mortality for proximal femur fractures seems overrated. Age and number of comorbidities significantly influence mortality rates. Surgery for hip fractures should take place as soon as reasonably possible, but delays up to 48 h are permissible without increasing mortality risk and should be used for optimizing preoperative patient status if necessary. There is no clear evidence that 24 h is a target that has been met to improve patient outcome and reduce mortality rates, and resources of our health care should be considered too.

## Data Availability

All data concerning the study is available.
